# S8-1 Health Promoting Sports Federations: theory, tools, case studies and future directions

**DOI:** 10.1093/eurpub/ckad133.038

**Published:** 2023-09-11

**Authors:** Chair(s):, Aurélie Van Hoye, Susanna Geidne

**Affiliations:** University of Limerick, Ireland; Örebro University

## Abstract

**Purpose:**

Beyond promoting their sports and contributing to elite success, sports federation have a broader health and sport for all promoting role, to include everyone in sport. The symposium has as objective to showcase theoretical grounds, case studies and tools to support health and physical activity promotion among international and national sports federation.

**Methods:**

The symposium will entail four presentation reflecting on different aspects of health and physical activity promotion. The first presentation will focus on theoretical grounds and guidelines created by 15 researchers and in collaboration with the World Health Organisation. The second presentation describes a case study among 51 national sports federation in France, to explain how they promote health and showcase the use of the indicators of health promoting sports federation indicators created by authors of the first presentation. The third presentation will describe a World Athletics project on the promotion of physical activity through competitions organisation and its stakes, as a practice-oriented example. The fourth presentation offer insights on the development and use of the “International and European Sport Organisations Activate Citizens” Capacity Building Framework, created through an Erasmus+ project, which entails a set of tools to empower international sports federation to promote sport for all.

**Results:**

After the four presentation, an open discussion will be engaged with the audience on the success and challenges, as well as a small interactive session on future directions for international and national sports federation to consider their health enhancing physical activity potential and fully exploit it.

**Conclusions:**

The symposium will equip the audience with theory, tools and example of practice, as well as reflect on how to move the research and practice agenda for health promoting sport federation.

